# Increased risk of hair loss with GLP-1 receptor agonists: A real-world multicenter TrinetX cohort study

**DOI:** 10.1016/j.jdin.2026.01.014

**Published:** 2026-02-09

**Authors:** Savanna I. Vidal, Yagiz Matthew Akiska, Mana Nasseri, Nikita Menta, Dillon Nussbaum, Colleen H. Cotton, Leslie Castelo-Soccio, Adam Friedman

**Affiliations:** a Department of Dermatology, George Washington University School of Medicine and Health Sciences, Washington, District of Columbia; b The Milken Institute School of Public Health, George Washington University, Washington, District of Columbia; c Division of Dermatology, Children's National Hospital, Washington, District of Columbia

**Keywords:** alopecia, alopecia areata, androgenetic, diabetes mellitus, type 2, drug-related side effects and adverse reactions, glp, glucagon-like peptide 1 receptor agonists, obesity, pharmacovigilance, telogeneffluvium

*To the Editor:* Glucagon-like peptide-1 receptor agonists (GLP-1RAs) are incretin-based therapies widely used for type 2 diabetes mellitus (T2DM) and obesity management. While their cardiovascular and metabolic benefits are well established, emerging reports suggest potential dermatologic adverse effects, particularly nonscarring hair loss (NSHL).^1^ Pharmacovigilance data and small cohort studies suggest a possible link between GLP-1RAs and hair loss, though evidence remains inconsistent and largely limited to post-marketing surveillance.^2^^,^^3^ Given the growing use of GLP-1RAs, further elucidating their dermatologic side effect profile and safety is essential.

This study used the TriNetX US Collaborative Network, a platform of aggregate de-identified electronic health record data from 67 US healthcare organizations, to evaluate the incidence and risk of NSHL in adults (18-89 years) and adolescents (12-17 years) treated with GLP-1RAs compared with matched controls from 2014 to 2024. Patients with ≥2 GLP-1RA prescriptions were included, while controls had ≥2 general medical encounters and no GLP-1RA exposure. Patients with confounding dermatologic, endocrine, nutritional, or systemic conditions were excluded (Supplementary Table I, available via Mendeley at https://data.mendeley.com/datasets/fhxs63v4s2/1). Propensity score matching accounted for age, sex, race, BMI, and T2DM status. NSHL outcomes, including telogen effluvium (TE), androgenetic alopecia (AGA), and alopecia areata (AA), were identified using ICD-10 codes. Follow-up for HL outcomes began 1 day after the index date and continued through 6- and 12-month intervals. Logistic regression estimated adjusted odds ratios (aORs) with 95% confidence intervals.

Among 547,993 matched adult GLP-1RA users and controls, cohorts were well-balanced (Supplementary Table II, available via Mendeley at https://data.mendeley.com/datasets/fhxs63v4s2/1). Between 2014 and 2024, the incidence of NSHL, TE, AGA, and AA increased in both GLP-1RA users and controls. Beginning around 2019, incidence curves for overall NSHL began to diverge, with GLP-1RA users showing consistently higher rates by 2023-2024. For TE and AGA, rates remained similar until 2021 to 2022 before rising more sharply among GLP-1RA users (Fig 1). Ten or fewer pediatric GLP-1RA users experienced each HL outcome of interest, precluding meaningful comparisons. At 6 months, GLP-1RAs were linked to a higher risk of AGA (aOR 1.62) and NSHL (aOR 1.26) (both *P* < .001) and increased TE risk (*P* = .18). At 12 months, risks increased for TE (aOR 1.76), AGA (aOR 1.64), and NSHL (aOR 1.40), all *P* < .001 (Fig 2).

**Fig 1:**
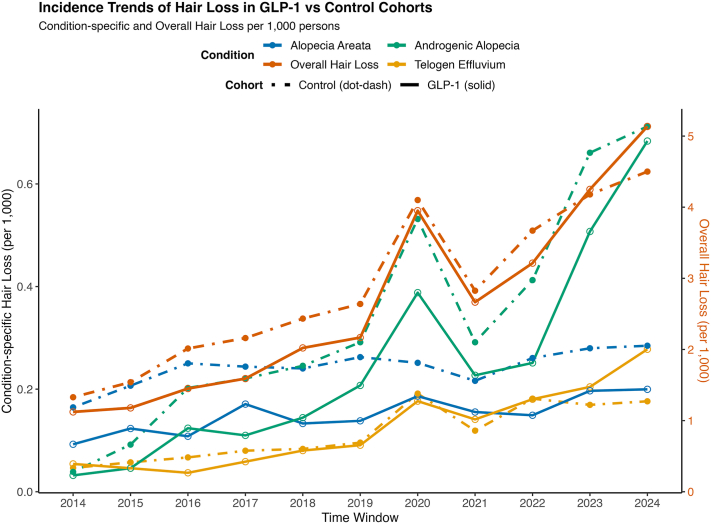
Annual incidence trends of hair loss among GLP-1RA users and controls, 2014-2024. Line graphs depict the temporal trends in the incidence of overall non-scarring hair loss and subtypes among individuals treated with GLP-1 receptor agonists compared with matched controls. The *left* y-axis represents condition-specific incidence proportions (telogen effluvium, alopecia areata, and androgenic alopecia) per 1000 persons. The *right* y-axis represents the overall incidence of non-scarring hair loss per 1000 persons. Solid lines indicate GLP-1 users, while *dot-dash lines* indicate control cohorts. Data are scaled using a dual y-axis for visibility of both overall and subtype-specific trends.

**Fig 2:**
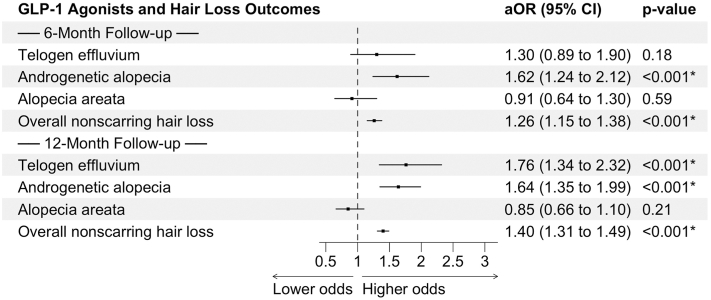
Association between GLP-1RA use and hair loss outcomes at 6 and 12 months. Forest plots displaying adjusted odds ratios (aORs) with 95% confidence intervals for the risk of TE, AGA, AA, and overall NSHL among GLP-1RA users compared with matched healthy controls at 6- and 12-month follow-up. Results show significantly increased odds of TE, AGA, and overall NSHL with GLP-1RA exposure, while no significant association was observed for AA.

In this matched cohort study, GLP-1RA use was associated with an increased risk of NSHL, especially TE and AGA, independent of demographic and clinical factors. TE showed a notable rise among GLP-1RA users, while AGA incidence increased in both groups, reflecting its progressive nature, with a slightly higher rise in GLP-1RA users possibly due to drug-related, metabolic, or surveillance effects. Likely mechanisms include rapid weight loss, insulin/insulin-like growth factor 1 signaling, androgen changes, and direct follicular effects.^4^ These findings align with prior studies linking alopecia to the GLP-1RAs semaglutide and tirzepatide.^3^^,^^5^ AA was consistently higher in controls, suggesting GLP-1RAs do not significantly affect autoimmune hair loss. In the pediatric subset, a small sample size limited analysis, highlighting barriers to access of GLP-1RAs in children.

Awareness of alopecia risk in patients on GLP-1RAs is critical for early detection, anticipatory guidance, and multidisciplinary care. Future research should examine underlying mechanisms, prospective monitoring, and pediatric outcomes to guide safe and informed GLP-1RA use.

## Conflicts of interest

None disclosed.
